# Azides and Porphyrinoids: Synthetic Approaches and Applications. Part 2—Azides, Phthalocyanines, Subphthalocyanines and Porphyrazines

**DOI:** 10.3390/molecules25071745

**Published:** 2020-04-10

**Authors:** Ana R. L. Araújo, Augusto C. Tomé, Carla I. M. Santos, Maria A. F. Faustino, Maria G. P. M. S. Neves, Mário M. Q. Simões, Nuno M. M. Moura, Sultan T. Abu-Orabi, José A. S. Cavaleiro

**Affiliations:** 1 LAQV-REQUIMTE, Department of Chemistry, University of Aveiro, 3810-193 Aveiro, Portugal; anararaujo@ua.pt (A.R.L.A.); actome@ua.pt (A.C.T.); cims@ua.pt (C.I.M.S.); faustino@ua.pt (M.A.F.F.); gneves@ua.pt (M.G.P.M.S.N.); msimoes@ua.pt (M.M.Q.S.); 2 CQE, Centro de Química Estrutural and IN—Institute of Nanoscience and Nanotechnology of Instituto Superior Técnico, Av. Rovisco Pais, 1049-001 Lisboa, Portugal; 3 Chemistry Department, Yarmouk University, Irbid 211-63, Jordan; dr.abuorabi@gmail.com

**Keywords:** azides, phthalocyanines, subphthalocyanines, porphyrazines, click chemistry, photodynamic therapy, photoinactivation

## Abstract

The reaction between organic azides and alkyne derivatives via the Cu(I)-catalyzed azide–alkyne cycloaddition (CuAAC) is an efficient strategy to combine phthalocyanines and analogues with different materials. As examples of such materials, it can be considered the following ones: graphene oxide, carbon nanotubes, silica nanoparticles, gold nanoparticles, and quantum dots. This approach is also being relevant to conjugate phthalocyanines with carbohydrates and to obtain new sophisticated molecules; in such way, new systems with significant potential applications become available. This review highlights recent developments on the synthesis of phthalocyanine, subphthalocyanine, and porphyrazine derivatives where CuAAC reactions are the key synthetic step.

## 1. Introduction

**Figure 1:**
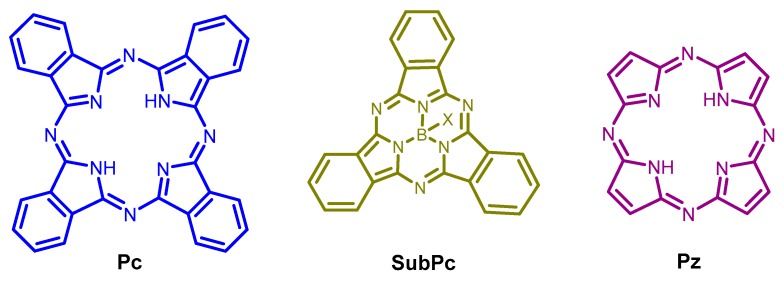
General structures of phthalocyanine (**Pc**), subphthalocyanine (**SubPc**) and porphyrazine (**Pz**).

This is Part 2 of a comprehensive review on “Azides and Porphyrinoids” [1]. Here, it will be presented the most relevant information about the chemistry of azides involving macrocycles such as phthalocyanines, subphthalocyanines, and porphyrazines (Figure 1) published in recent years, mainly since 2015.

Phthalocyanines (Pcs) are aromatic macrocycles that exhibit particularly interesting photophysical, photochemical, semiconducting, and photoconducting properties. Such compounds find applications in different fields, mainly in photodynamic therapy (PDT) of tumors, in electrocatalysis, as semiconducting and non-linear optical devices, as chemosensors, and as dyes in solar cells [2,3,4,5,6,7]. The other two types of heterocycles considered in this review, the subphthalocyanines and the porphyrazines, although less studied than Pcs, are also important targets in organic synthesis. Subphthalocyanines are homologues of phthalocyanines, being constituted by three isoindole subunits with a 14 π-electron aromatic core. These compounds are being explored in different fields of applications (e.g., chemical sensors, electronic molecular devices). Porphyrazines (or tetraazaporphyrins) also merit a special attention from the scientific community due to their potential role as PDT photosensitizers, as chemical sensors and in the development of molecular electronic devices, Langmuir-Blodgett films, ladder polymers and in biomimetic chemistry [8].

Most of the publications discussed in this review involve the formation of phthalocyanine, subphthalocyanine and porphyrazine derivatives bearing 1,2,3-triazolyl groups generated from Cu(I) catalyzed azide-alkyne cycloaddition (CuAAC) reactions, one of the most used and useful type of click reactions.

## 2. Phthalocyanines in CuAAC Reactions

The CuAAC approach is a versatile and efficient strategy to synthesize new phthalocyanine derivatives and to incorporate phthalocyanine units on the surface of several nanomaterials, such as carbon materials (e.g., graphene oxide, carbon nanotubes), silica nanoparticles, gold nanoparticles, and quantum dots (e.g., CdSe/ZnS), as well as in polymers, biomolecules, and electrodes [9,10]. As discussed in the following sections, this approach has led to the development of improved and multifunctional systems based on Pcs with electrical and optical properties adequate for photovoltaic applications, sensing, photodynamic therapy, etc.

### 2.1. Phthalocyanines and New Carbon Nanomaterials

**Scheme 1:**
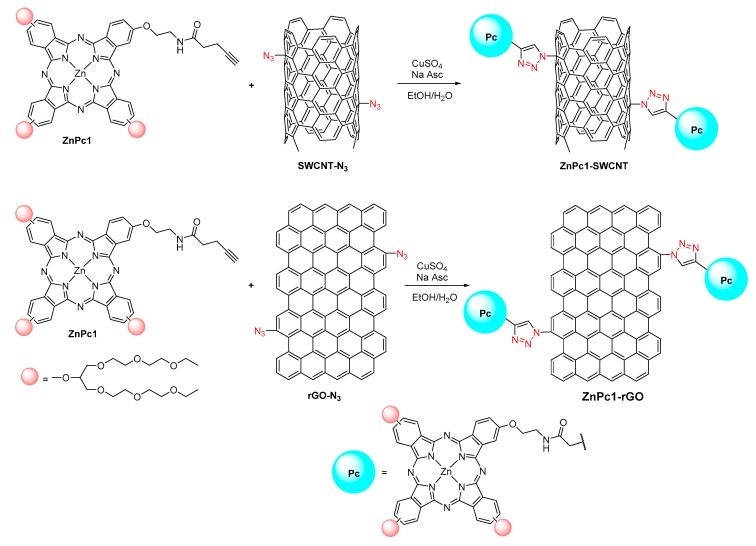
Synthetic strategy used to immobilize **ZnPc1** on single-walled carbon nanotubes (**SWCNTs**) and on reduced graphene oxide (**rGO**).

Innovative phthalocyanine carbon nanomaterials with improved electronic, chemical and mechanical properties have been obtained in recent decades. In this context, in 2016, Kadem et al. described the covalent attachment of the asymmetrical zinc(II) phthalocyanine **ZnPc1**, functionalized with a terminal alkyne unit, to the surface of azido-substituted single-walled carbon nanotubes (SWCNTs) and to azido-substituted reduced graphene oxide (rGO), using CuSO_4_·5H_2_O as catalyst and sodium ascorbate (Na Asc) as reducing agent (Scheme 1) [11].

The obtained hybrids were characterized by using adequate techniques (e.g., FTIR, Raman spectroscopy, UV-Vis absorption). From absorption spectra and cyclic voltammetry studies it was found that the covalent attachment of **ZnPc1** to SWCNTs or to rGO resulted in band gaps’ values lower than the ones found in free **ZnPc1** (1.62 eV *versus* 1.77 eV) or in the hybrids obtained from its non-covalently attachment to SWCNTs or rGO (*ca.* 1.68 eV). The electron transfer process across the substituted ZnPc to the SWCNTs or rGO is favored by the presence of the triazole lone pair electrons. The studies have also shown an improvement in the electrical conductivity for both types of hybrids (covalently and non-covalently linked) when compared with the pure phthalocyanine film, being particularly relevant to the increase observed for the hybrid covalently linked to SWCNTs (218.6 mS/cm *versus* 11.4 mS/cm). The results obtained are contributions for the relevant area of photovoltaic applications.

**Scheme 2:**
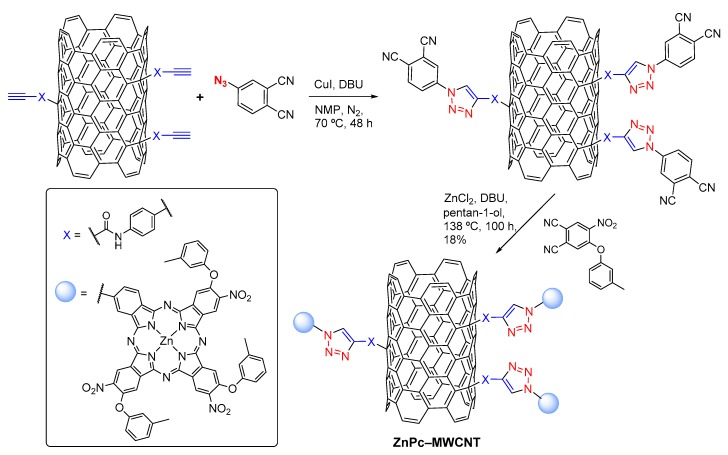
Synthetic strategy used to obtain the hybrid **ZnPc-MWCNT**.

In 2018, Yang and co-workers [12], having in mind the synthesis of novel organic-inorganic hybrid materials with a high content of grafted phthalocyanine derivative on the surface of multi-walled carbon nanotubes (MWCNT), reported the synthesis of **ZnPc-MWCNT** assemblies as illustrated in Scheme 2. The hybrid was prepared in two steps that started with the reaction of an acetylene-functionalized MWCNT with 4-azidophthalonitrile in the presence of copper(I) iodide, 1,8-diazabicyclo[5.4.0]undec-7-ene (DBU) and l-methyl-2-pyrrolidone (NMP). The second step consisted in the reaction of the formed phthalonitrile-modified MWCNT with 4-nitro-5-(*m*-tolyloxy)phthalonitrile. The covalent conjugation of the phthalocyanine to the MWCNT was confirmed by FTIR, TEM, SEM, AFM, UV-Vis and fluorescence spectra. It was verified that the solubility of **ZnPc-MWCNT** in tetrahydrofuran (THF) was significantly improved when compared with the one of the MWCNT.

**Scheme 3:**
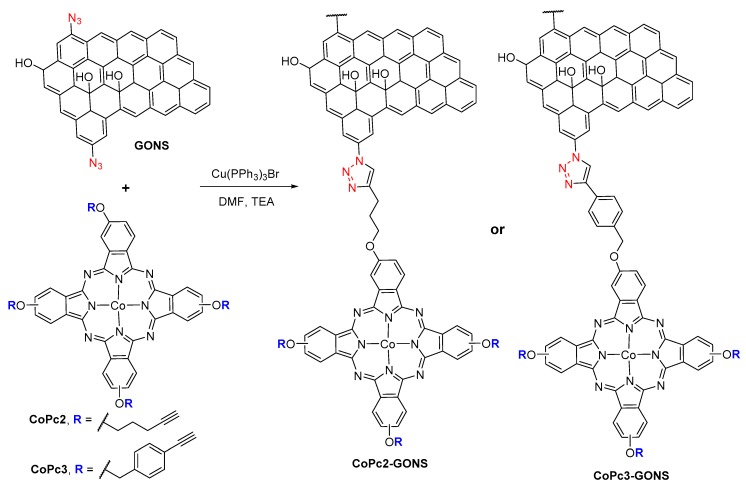
Synthetic methodology used to prepare **CoPc2-GONS** and **CoPc3-GONS** assemblies.

In 2019, Mpeta and Nyokong reported that the reaction of reduced graphene oxide nanosheets bearing azido groups (GONS) with alkynyl-substituted cobalt(II) phthalocyanine derivatives (**CoPc2** and **CoPc3**) allowed to obtain **CoPc2-GONS** and **CoPc3-GONS** assemblies (Scheme 3) [13]. The cycloaddition reaction was performed in *N*,*N*-dimethylformamide (DMF) in the presence of Cu(PPh_3_)_3_Br and trimethylamine. The electrocatalytic activity of the resulting triazole hybrids was evaluated in the oxidation of 2-mercaptoethanol, a compound with an important role in the metabolism and cellular homeostasis and used in corrosion inhibition processes. The study showed that the electrode obtained with **CoPc3-GONS** (the alkyne group is separated from the phthalocyanine ring by an aliphatic group and a benzene ring) is a promising electrochemical sensor for 2-mercaptoethanol, showing the best catalytic activity when compared with the one with **CoPc2-GONS**. Additionally, the same hybrid exhibited the highest sensitivity and a lower limit of detection than other sensors reported in the literature.

### 2.2. Phthalocyanine-Silica Nanoparticles

In recent years, silica nanoparticles (SiNPs) have received significant attention due to their chemical inertness, non-toxicity, optical transparency, and excellent thermal stability. SiNPs have been widely used in catalysis, chemical processes in industry, removal of metal ions, encapsulation of organic light-emitting diodes and also as nanocarriers for photosensitizers [14].

**Scheme 4:**
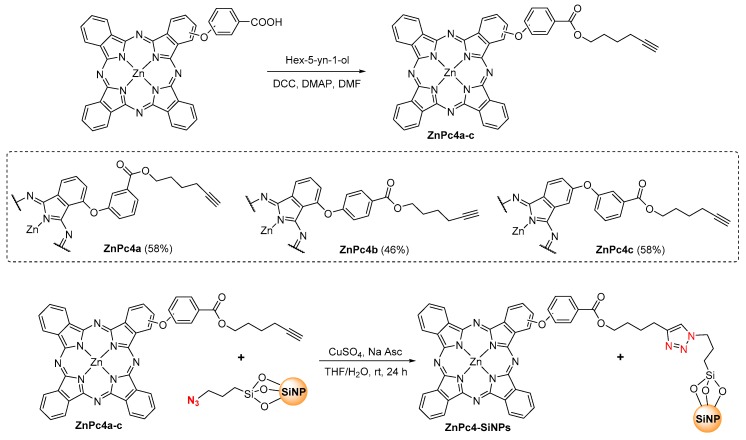
Synthesis and conjugation of **ZnPc4a**–**c** with azide-functionalized silica nanoparticles.

The CuAAC reaction has been used for the modification of silica nanoparticles and in their subsequent conjugation with organic molecules, namely with Pcs. For example, Nyokong and co-workers described the synthesis of the mono-substituted zinc(II) phthalocyanines **ZnPc4a**–**c** bearing terminal alkynyl units and their conjugation with silica nanoparticles functionalized with azido groups (Scheme 4) [15]. The alkynyl-substituted phthalocyanines were prepared by reacting the adequate carboxyphenoxyphthalocyanines with hex-5-yn-1-ol in the presence of the coupling agents *N*,*N’*-dicyclohexylcarbodiimide (DCC) and 4-(dimethylamino)pyridine (DMAP); the conjugation with the azido-nanoparticles was performed in THF/H_2_O, at room temperature, in the CuAAC conditions (Scheme 4).

From the photophysical properties of the hybrids and of the starting materials, it was observed that the esterification of the zinc(II) phthalocyanines with hex-5-yn-1-ol increases the fluorescence quantum yields and the fluorescence is maintained after the grafting process. The triplet quantum yields of **ZnP4a**–**c** (*ca.* 0.65) increased significantly when compared to those of the precursor phthalocyanine complexes (0.45), but decreased after the grafting to the SiNPs. On the other hand, the triplet lifetimes of all hybrids increased in comparison to the lifetimes of the non-immobilized ZnPcs and this behavior was justified by considering a possible protection provided by the polymeric silica nanoparticles to the phthalocyanines in the triplet state [15].

**Scheme 5:**
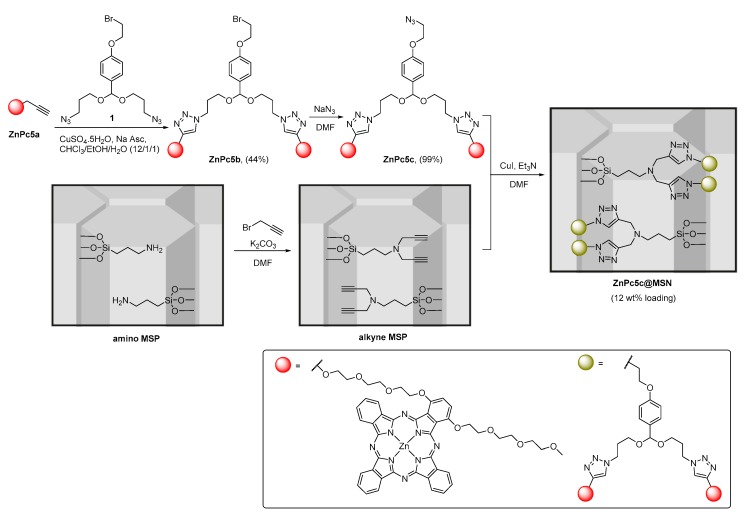
Synthetic strategy used to prepare the triazole **ZnPc5c@MSN**, the precursors **ZnPc5a**–**c** and the alkyne-silica nanoparticles.

In 2017, Wong and co-workers selected the CuAAC approach in different steps of the synthetic strategy to develop the triazole photoactive nanoparticles **ZnPc5c@MSN** (Scheme 5) [16]. The approach involved the conjugation of mesoporous silica nanoparticles (MSN), functionalized with alkyne units, with the phthalocyanine dimer **ZnPc5c** containing an azido terminal group. This dimer was obtained by reacting the previously described [17] alkynyl **ZnPc5a** with the acetal linker (obtained from 4-(2-bromoethoxy)benzaldehyde and 3-azidopropan-1-ol) under CuAAC conditions, followed by the reaction of the obtained **ZnPc5b** with sodium azide. The alkyne-silica nanoparticles were prepared by reacting the amino mesoporous silica nanoparticles (obtained according with literature data [18]) with 3-bromopropyne (Scheme 5). The conjugation of **ZnPc5c** with the alkyne-functionalized MSN was performed in DMF in the presence of CuI and Et_3_N. The study showed that the fluorescence and the photosensitizing efficiency strongly quenched in the native triazole **ZnPc5c@MSN** form was restored in acidic PBS (pH 5.5) or after its internalization in human colon adenocarcinoma HT29 cells. The release of **ZnPc** units from the acetal linker under the acidic conditions justified the high intracellular fluorescence and the photocytotoxicity observed even in the in vivo studies. This behavior was not observed in analogue systems with a non-cleavable dimer, and that suggested that this nanosystem was a promising photosensitizing agent for PDT.

### 2.3. Phthalocyanine-Gold Nanoparticles

**Scheme 6:**
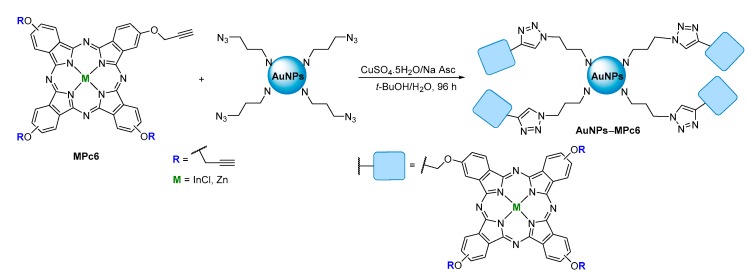
Preparation of nanocomposites **AuNPs-ZnPc6** and **AuNPs-InPc6**.

Gold nanoparticles (AuNPs) are one of the most significant and stable groups of metal nanoparticles with potential application in non-linear optical devices for protecting light-sensitive materials, amongst other applications [19]. With the aim of improving the non-linear optics of Pcs, in 2017, Bankole and Nyokong selected the CuAAC approach to obtain conjugates of AuNPs with phthalocyanines [20]. The nanocomposites **AuNPs-ZnPc6** and **AuNPs-InPc6** (Scheme 6) were obtained using azide derivatized gold nanoparticles and the indium(III) and zinc(II) phthalocyanine complexes **InPc6** and **ZnPc6** bearing alkynyl units. The azide derivatized gold nanoparticles were obtained by partial ligand exchange of tetraoctylammonium bromide moieties with 3-azidopropylamine via N-Au bonds and the coupling of both components was performed in the presence of CuSO_4_·5H_2_O and sodium ascorbate (Scheme 6) [20]. Photophysical studies showed that the obtained nanocomposites had lower fluorescence quantum yields and lifetime values than those obtained for the unconjugated phthalocyanines; the quenching of the excited singlet states was justified as being due to the heavy atom effect.

Another gold nanomaterial that has been conjugated with phthalocyanines via the CuAAC reaction is the nanoporous gold (npAu). Recently, it has been shown that npAu can be used as a support for biomolecules in biosensors development studies, also as a catalyst for the oxidation of small molecules and as a material with plasmonic properties useful for optical sensing. This monolithic nanomaterial is known to exhibit a high surface area and so it can be easily functionalized with a thiol or disulfide groups in the context of self-assembled monolayers (SAMs) [21].

**Scheme 7:**
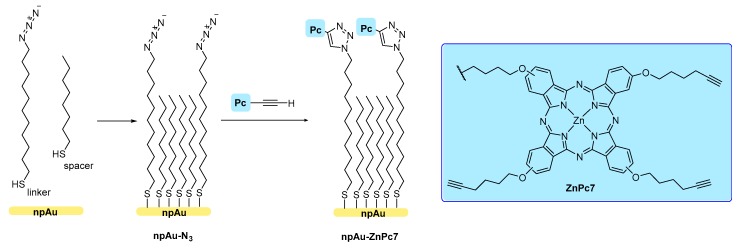
Synthetic strategy used for the preparation of **npAu-N_3_** and **npAu-ZnPc7**.

In 2014, Wittstock and co-workers [22] reported the development of a npAu composite (**npAu-ZnPc7**) which demonstrated to have photocatalytic features; the synthesis was performed by coupling the Zn(II) complex of tetrakis(hex-5-ynyloxy)phthalocyanine (**ZnPc7**) with the nanoporous gold material functionalized with azide units (**npAu-N_3_**) (Scheme 7). In the formation of the self-assembled monolayer (SAM) on the nanoporous gold material, the authors used 11-azidoundecane-1-thiol as the azide linker and octane-1-thiol as a shorter ‘spacer’ in order to avoid mutual steric hindrances between the active molecules. The cycloaddition reaction between both components was performed in a mixture of THF and water in the presence of Cu[TBTA]PF_6_ and hydroquinone. The photocatalytic efficacy of the nanocomposite was evaluated in the oxidation of (*S*)-(−)-citronellol. A conversion of 40% was reached while no reactivity was detected when the same photocatalyst was immobilized on the planar gold surface or when it was dissolved in the reaction solution. The authors have commented that this better performance is probably associated to the material nanoporosity and consequently to an increase in the surface area and also to the npAu electronic and optical properties namely the plasmonic effect.

In 2019, the same group used a similar SAM approach to immobilize **ZnPc7** on npAu functionalized with a short azide linker (azidohexylthiolate groups) aiming to improve the interaction between the ZnPc and the npAu surface [23]. The catalytic efficacy of the resulting hybrid system was evaluated in the photooxidation of 1,3-diphenylisobenzofuran (DPBF), a well-known ^1^O_2_ quencher, at different irradiation wavelength; the highest photocatalytic activity was observed at 700 nm. The hybrid showed higher photocatalytic activity than the separated components (**ZnPc7** in solution or the npAu). The synergism between the npAu surface plasmons and the ZnPc during the DPBF photooxidation was confirmed by the improvement of nearly an order of magnitude in the photooxidation activity only when both absorption sites were irradiated simultaneously.

### 2.4. Phthalocyanine-Quantum Dots Hybrids

**Scheme 8:**
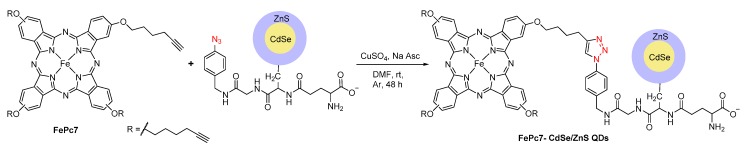
Schematic representation of the synthesis of **FePc7-CdSe/ZnS** quantum dots (**QDs**).

Quantum dots (QDs) are semiconductor nanocrystals that alone or after being adequately functionalized can be used in the electrocatalytic analysis of several analytes. Under this context, Nxele and Nyokong reported the access to the hybrid **FePc7-CdSe/ZnS QDs** through the conjugation of the azide-functionalized CdSe/ZnS QDs with the Fe(II) tetrakis(hex-5-ynyloxy)phthalocyanine (**FePc7**) using CuSO_4_·5H_2_O and sodium ascorbate in the cycloaddition process (Scheme 8) [24]. The resultant hybrid was used in the electrocatalytic detection of the pesticide paraquat; the latter is well-known by its toxicity and resistance to biodegradation. The electrocatalytic behavior of the clicked sensor (**FePc7-CdSe/ZnS**) was studied using various voltammetry techniques and scanning electrochemical microscopy; a lower limit of detection (LOD = 5.9 mmol mL^−1^) compared to the action of other sensors for paraquat was found.

### 2.5. Phthalocyanine-Carbohydrate Conjugates

Phthalocyanines (Pcs) are potential near infra-red photosensitizers to be used to treat bacterial infections and cancers. However, their clinical application is hindered by their poor solubility in biological fluids. In fact, the poor water solubility and aggregation of Pcs in that medium are typically high and recent research has been focused on the development of water-soluble phthalocyanines [25]. In such way, carbohydrates are an attractive type of biomolecules that can be conjugated with phthalocyanines to improve their water solubility. Several examples based on the attachment of phthalocyanines to carbohydrates are known.

**Scheme 9:**
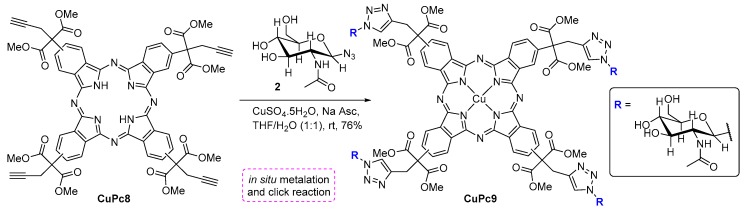
Synthesis of the glycosylated **CuPc9**.

In 2014, Yilmaz and co-workers reported the synthesis of the glycosylated copper(II) phthalocyanine derivative **CuPc9**. This compound was obtained from the CuAAC reaction of the alkynyl-substituted phthalocyanine **CuPc8** with 2-acetamido-2-deoxy-β-D-glucopyranosyl azide **2** (Scheme 9) [26]. Phthalocyanine **CuPc8** was obtained by cyclotetramerization of dimethyl (3,4-dicyanophenyl)propargylmalonate, in pentan-1-ol and in the presence of DBU [27]. The 1,3-dipolar cycloaddition took place in THF, at room temperature, in the presence of CuSO_4_·5H_2_O and sodium ascorbate. The photophysical characterization of **CuPc9** showed that the coordination of the phthalocyanine inner core with copper(II) was responsible for a drastic decrease in the photoluminescence intensity and in the quantum yield; the increase in the non-radiative processes and decreasing the fluorescence emission was justified by considering the energy transfer from the excited state of the ligand to the metal ion.

**Scheme 10:**
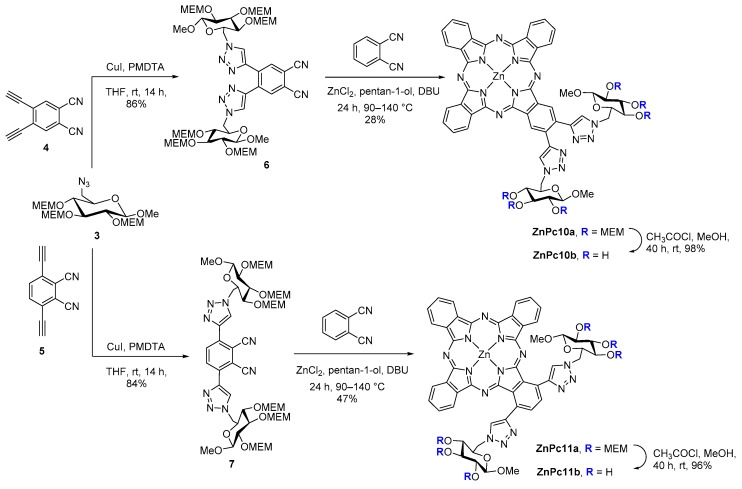
Synthetic strategy used in the preparation of **ZnPc10a**/**10b** and **ZnPc11a**/**11b**.

In the same year, Bächle and co-workers, aiming to obtain “third generation PSs” with adequate amphiphilicity and specific uptake by tumor cells due to sugar moieties, prepared the AB_3_-saccharide zinc(II) phthalocyanines **ZnPc10b** and **ZnPc11b** using phthalonitrile and the glycoconjugated phthalonitriles **6** and **7**; the latter ones were obtained from adequate alkyne-substituted phthalonitriles **4** and **5** and the protected 6-azido-glucopyranoside **3** (Scheme 10) [28]. The co-tetramerization of the glycoconjugated phthalonitrile precursors **6** and **7** with phthalonitrile in pentan-1-ol in the presence of zinc(II) chloride, afforded **ZnPc10a** and **ZnPc11a**, which after deprotection of the sugar units gave rise to **ZnPc10b** and **ZnPc11b**. The authors commented on the significance of the 2-methoxyethoxymethyl (MEM) group as the protecting group of the sugar units. That was due to the stability under harsh basic cyclotetramerization conditions and to the facile removal under mild acidic conditions. The conjugates exhibited well-defined UV-Vis spectra in DMSO (ε_max_ > 10^5^ M^−1^ cm^−1^ and absorption maxima λ > 680 nm) but broadened and diminished Q-bands were observed in water and that was probably due to aggregation. Interestingly, the formation of *J*-type aggregated forms seems to occur with **ZnPc10b** since an unexpected bathochromic shifted in the Q band was observed.

**Scheme 11:**
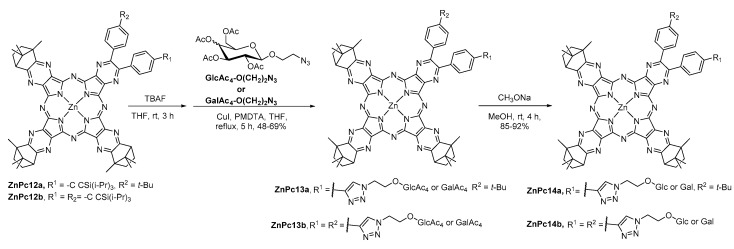
Synthesis of glycosylated azaphthalocyanines Z**nPc14a**,**b**.

Park and co-workers also reported the synthesis of the more hydrophilic A_3_B-type azaphthalocyanine zinc(II) complexes **ZnPc14a** and **ZnPc14b**. These Pcs contain three bulky three-dimensional bornane groups and one or two β-D-glucosyl or β-D-galactosyl moieties [29]. The authors have envisaged that the presence of the bornane groups could prevent the axial or peripheral approach between photosensitizers (PSs) without altering their optical properties. The coupling of the azaphthalocyanine complexes bearing one or two alkyne units (obtained from Z**nPc12a** and **ZnPc12b** after deprotection with TBAF) with the adequate azide carbohydrates was performed in the presence of CuI and PMDTA (Scheme 11). The structures of the expected compounds were confirmed using adequate spectroscopic techniques and the photophysical studies confirmed that in DMSO all compounds were monomeric, but aggregation was detected in DMSO/water.

**Scheme 12:**
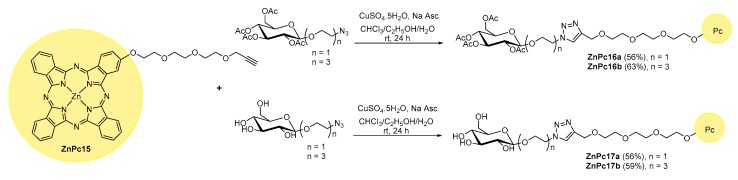
Synthetic strategy used to prepare the glycosylated phthalocyanines **ZnPc16** and **ZnPc17**.

Liu and co-workers also used azide-substituted glucose derivatives to prepare the acetylated glucosyl phthalocyanines **ZnPc16a** and **ZnPc16b** and the glucosyl ones **ZnPc17a** and **ZnPc17b** using the propargylated phthalocyanine **ZnPc15** (Scheme 12) [30]. The cycloaddition reaction was done in both cases in the presence of CuSO_4_·5H_2_O and sodium ascorbate, at room temperature, affording the expected conjugates **ZnPc16** and **ZnPc17**.

The biological studies showed that compounds **ZnPc17a** and **ZnPc17b** have a specific affinity to MCF-7 breast cancer cells when compared with HELF normal cells; furthermore, both compounds are localized in the lysosome and exhibit high photocytotoxicity towards MCF-7 cells.

**Scheme 13:**
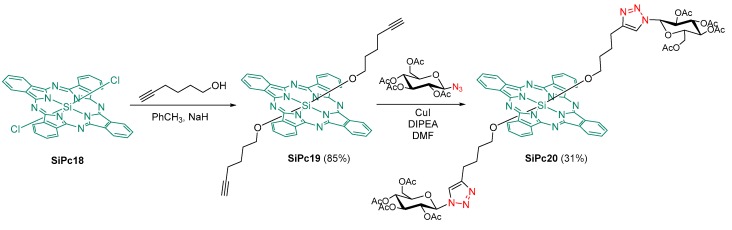
Synthetic strategy used to prepare axial carbohydrates **ZnPc20**.

A silicon(IV) phthalocyanine bearing two axial sugar units **SiPc20** was synthesized as illustrated in Scheme 13. Using a base-catalyzed ligand exchange approach, the silicon(IV) phthalocyanine dichloride **SiPc18** in the presence of hex-5-yn-1-ol afforded the silicon dialkyne **SiPc19** (Scheme 13) [31]. The last step involved the CuAAC reaction of the dialkynyl **SiPc19** and azido-2,3,4,6-tetra-*O*-acetyl-β-D-glucopyranose (GlcN_3_). This reaction was performed in DMF in the presence of copper(I) iodide and *N*,*N-*diisopropylethylamine (DIPEA) affording the **SiPc20** in 31% yield. Other sugar-azide derivatives, including those with galactosyl, xylosyl and mannosyl units, have also been used by the same group.

**Figure 2:**
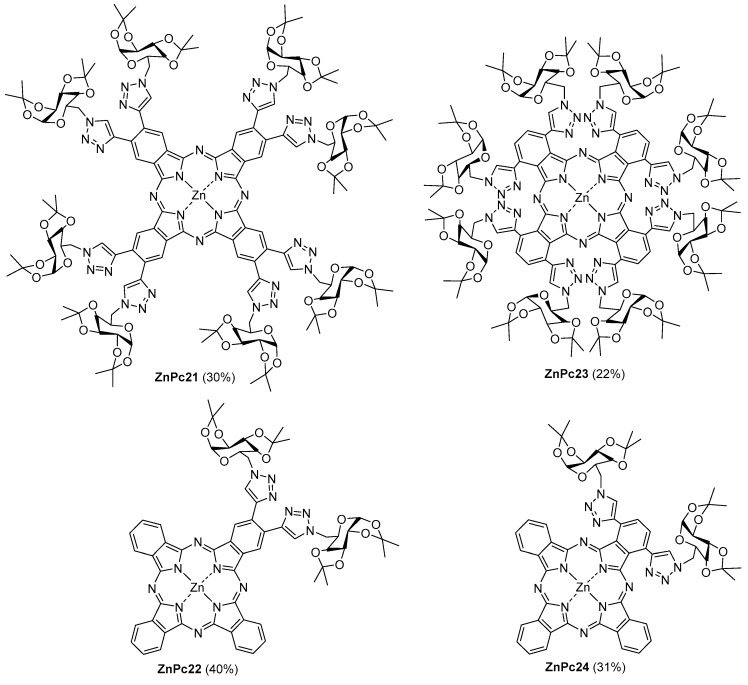
Structures of the galactoconjugated **ZnPc21**–**24**.

In 2019, Ziegler and co-workers reported the synthetic access to the four galactoconjugated zinc(II) phthalocyanines **ZnPc21–24** (Figure 2) using phthalonitrile and the adequate galactophthalonitrile derivative or just the galactophthalonitrile derivatives [32]. In the access to the galactophthalonitriles the approach was similar to the one previously reported by the same group but now using the 6-azido-1,2:3,4-di-*O*-isopropylidene-α-D-galactopyranose and the adequate bis-alkynyl phthalonitriles **6** and **7** (see Scheme 10). These glycoconjugated zinc(II) phthalocyanines in DMSO showed Q bands in the red visible light region (679–737 nm) with molar absorption coefficients (ε_max_) higher than 10^5^ M^–1^ cm^–1^. From the spectroscopic behavior in solution it was concluded that the synthesized phthalocyanines form *J*-aggregates.

**Scheme 14:**
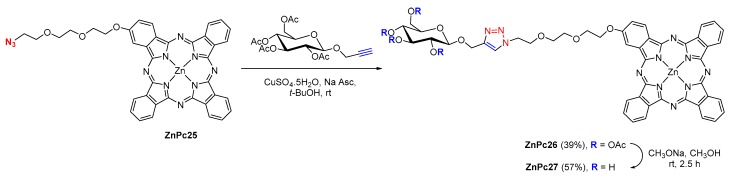
Synthetic strategy used to prepare **ZnPc27**.

Also in 2019, Uruma and co-workers reported the synthesis of the glucosylated phthalocyanine **ZnPc27** and its photodynamic action towards QR-32 and QRsP-11 cell lines (Scheme 14) [33]. The authors selected glucose functionalized with a propargyl unit and the zinc(II) phthalocyanine **ZnPc25** bearing an azido alkoxy group as reagents. **ZnPc26** has been obtained and after deprotection with sodium methoxide in methanol gave rise to **ZnPc27**. In the photocytotoxicity assays, **ZnPc27** caused apoptosis in QR-32 and QRsP-11 cell lines of rodent; a stronger photocytotoxicity against QR-32 cells than to QRsP-11 cells has been observed.

### 2.6. CuAAC Reactions Involving Phthalocyanines and Polymers

As previously mentioned, the solubility of phthalocyanines in various solvents is critical for their application in different fields. In recent years the possibility to organize phthalocyanines by using polymeric systems appeared to be an effective approach.

**Scheme 15:**
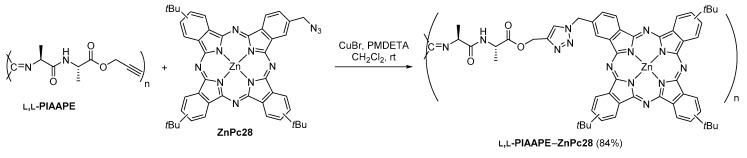
Synthetic strategy used for the immobilization of **ZnPc28** in **l,l-PIAAPE**.

Torres and co-workers described the immobilization of a Pc on a polymer by using the CuAAC reaction (Scheme 15) [34]. The authors used the helical rigid rod alkynyl-terminated polyisocyanide **L,L-PIAAPE** and the azide phthalocyanine **ZnPc28**; the reaction was performed in dichloromethane in the presence of copper(I) bromide and an excess of PMDETA at room temperature. The expected polymer **L,L-PIAAPE-ZnPc28** was obtained in 84% yield. From UV-Vis, fluorescence, and circular dichroism studies, it was confirmed that the phthalocyanine attachment did not affect the rod-like helical arrangement of the starting polymer.

**Scheme 16:**
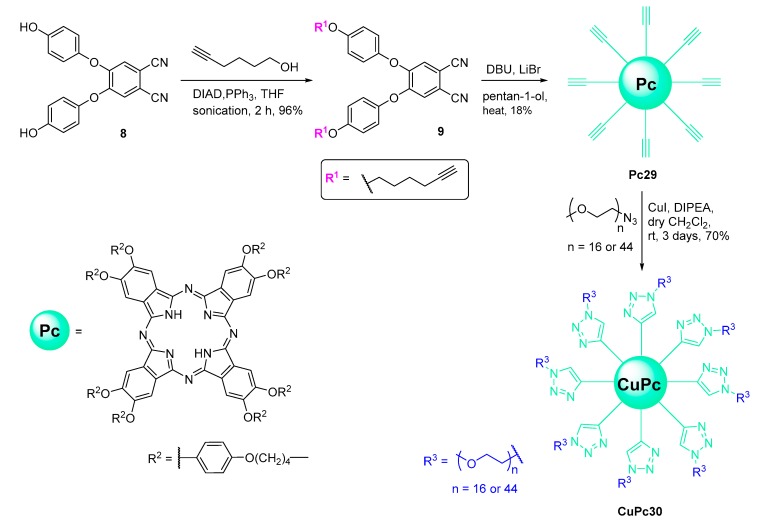
Synthesis of **CuPc30** with different chain lengths of monomethyl ether polyethylene glycol (mPEG).

Haddleton and co-workers combined the Mitsunobu reaction and the CuAAC approach to prepare the octasubstituted copper(II) phthalocyanines **CuPc30** with two different chain lengths of monomethyl ether polyethylene glycol (mPEG, 750–2000 Da) (Scheme 16) [35]. The alkyne-terminated phthalonitrile **9** was prepared from 4,5-bis(4-hydroxyphenoxy)phthalonitrile **8** using commercially available hex-5-yn-1-ol, triphenyl phosphine and diisopropyl azodicarboxylate (DIAD) according to the Mitsunobu conditions, giving rise to the “clickable” metal free **Pc29** after the cyclotetramerization step. Its conjugation with the two different azido monomethyl ether polyethylene glycol chains was performed in the presence of CuI and DIPEA affording **CuPc30**.

The PEGylated copper phthalocyanine polymers obtained proved to be highly soluble in a range of organic solvents (e.g., chloroform, dichloromethane, THF) with no evidence of aggregation. In water, the presence of dimeric species was detected by UV-Vis spectra with a minor contribution of monomeric species. From the DSC studies it was observed that the PEGylated **CuPc30** with the shorter chain (n = 16) exhibited a significant difference in thermal behavior when compared with commercial mPEGs and the authors have suggested that tunable thermal properties can be achieved with these systems by incorporating other mPEGs with different chain lengths.

**Scheme 17:**
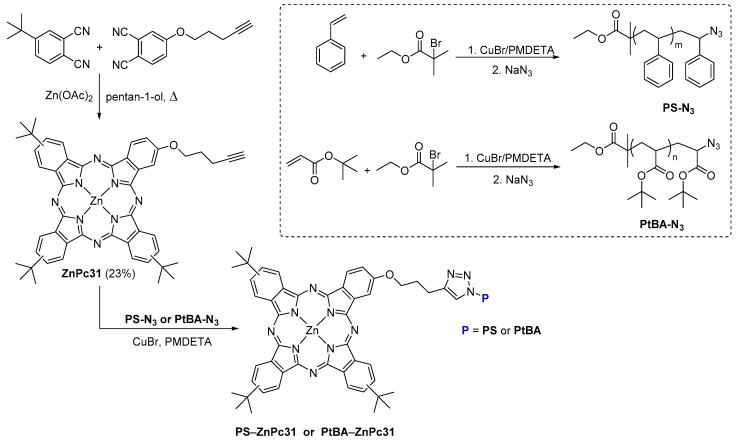
Synthesis of polymers **PS-ZnPc31** and **PtBA-ZnPc31** and of the required precursors **ZnPc31**, **PS-N_3_**, and **PtBA-N_3_**.

In 2014, Şen and co-workers reported that the conjugation of the unsymmetrically substituted zinc(II) phthalocyanine **ZnPc31**, with three *tert-*butyl groups and a terminal alkynyl moiety with the azido-terminated polystyrene **PS-N_3_** and poly(*tert*-butyl acrylate) **PtBA-N_3_** afforded the polymers **PS-ZnPc31** and **PtBA-ZnPc31** (Scheme 17) [36]. **ZnPc31** was obtained through the statistical condensation of 4-*tert*-butylphthalonitrile and 4-(pent-4-ynyloxy)phthalonitrile in the presence of zinc(II) acetate and the polymers were obtained using the atom transfer radical polymerization (ATRP) approach in the presence of PMDETA/CuBr, followed by the reaction of the obtained bromo end-functionalized polymers with NaN_3_.

The electrochemical studies allowed to conclude that conjugation of the **ZnPc31** to the polymers increased the chemical stability of the complex but decreased the electrochemical reversibility during redox reactions. However, the presence of polymer did not affect the spectroelectrochemical and electrocolorimetric responses of **ZnPc31**.

**Scheme 18:**
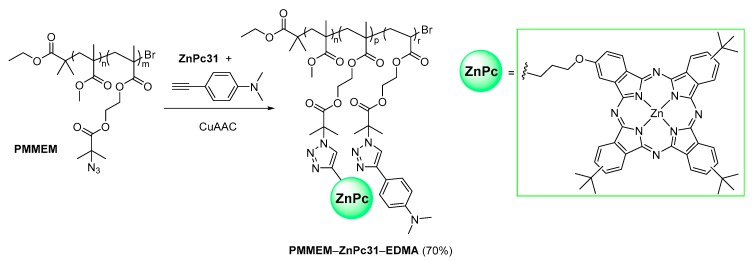
Synthesis of **PMMEM-ZnPc31-EDMA**.

A double click reaction was used to incorporate phthalocyanine **ZnPc31** and 4-ethynyl-*N*,*N*-dimethylaniline in the azido-functional poly(methyl methacrylate-*co*-2-(2-bromoisobutyryloxy)ethyl methacrylate) copolymer (PMMEM) (Scheme 18) [37]. The obtained copolymer **PMMEM-ZnPc31-EDMA** was crosslinked in the presence of the liquid crystal mesogen 4’-(octyloxy)-4-biphenylcarbonitrile by ultraviolet irradiation using benzophenone as initiator and ethylene glycol dimethacrylate as difunctional crosslinker. The resulting polymer-dispersed liquid crystal film was characterized using differential scanning calorimetry and polarized optical microscopy.

**Scheme 19:**
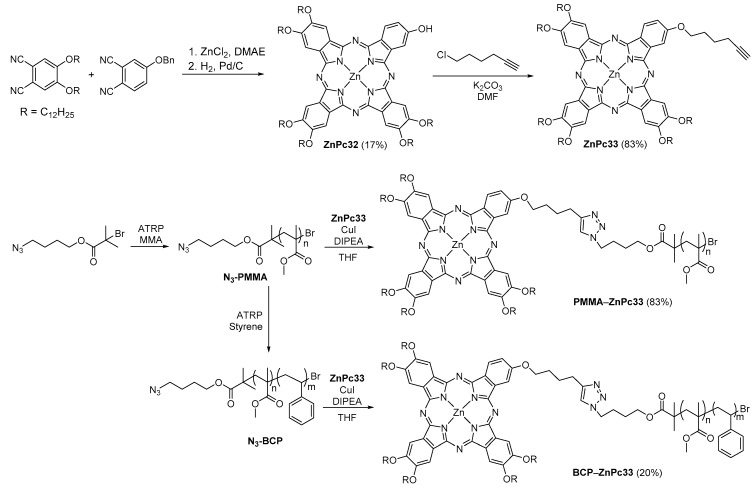
Synthesis of polymers **PMMA-ZnPc33** and **BCP-ZnPc33** and of their precursors.

Aimi and co-workers selected **ZnPc32** to be assembled to the azide-terminated poly(methyl methacrylate) (**N_3_-PMMA**) and to the azide-terminated block copolymer (**N_3_-BCP**) to afford the hybrid systems **PMMA-ZnPc33** and **BCP-ZnPc33** (Scheme 19) with potential applications in optoelectronic devices [38]. Phthalocyanine **ZnPc33** was obtained from the reaction of 6-chlorohex-1-yne with the hydroxyphthalocyanine **ZnPc32**; this template resulted from the tetracyclization of 4,5-bis(dodecyloxy)phthalonitrile with 4-benzyloxyphthalonitrile in the presence of ZnCl_2_, followed by cleavage of the benzyl group with H_2_/Pd/C. The final CuAAC reaction was performed in the presence of CuI and DIPEA in THF. The resulting Pc-containing polymer films exhibited a cylindrical morphology in which the Pc units showed π-π interactions inside the confined PMMA cylinders. The authors commented that these results can be explored in the development of organic photovoltaic (OPV) devices and organic field effect transistors (OFETs).

**Figure 3:**
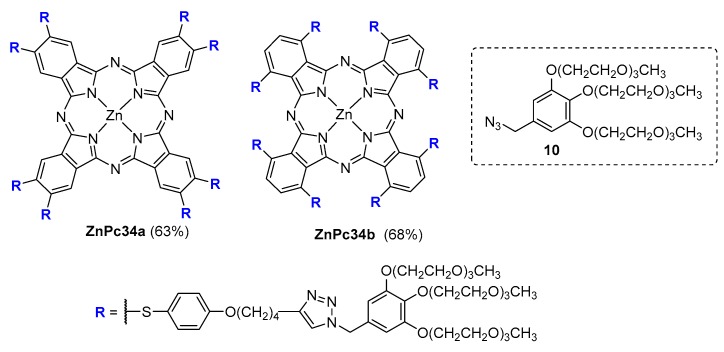
Structures of the dendritic zinc(II) Pcs **ZnPc34a** and **ZnPc34b**, and of the azide **10** used in their preparation.

McGrath and co-workers described the synthesis and the antimicrobial activity of the two dendritic water-soluble zinc(II) phthalocyanines **ZnPc34a**,**b** where the triethylene glycol (TEG) moieties where introduced by coupling the azide **10** bearing three TEG [39] units with the adequate alkynyl-substituted zinc phthalocyanines in the presence of CuI and DIPEA (Figure 3) [40].

The aggregation studies performed with **ZnPc34b** have shown that it is significantly less aggregated in aqueous media than the peripherally substituted isomer **ZnPc34a** and its Q band is about 80 nm red-shifted when compared with that of **ZnPc34a**. Both ZnPcs showed no dark toxicity against bacteria and yeast, at 10 μM concentration, but upon irradiation (400–850 nm) both demonstrated to be phototoxic to *Acinetobacter baumannii* (99.9999% cell inactivation with **ZnPc34b**) and to *Pseudomonas aeruginosa* (90% cell inactivation).

**Figure 4:**
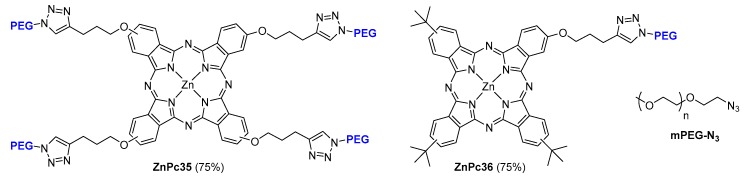
Structures **ZnPc35** and **ZnPc36** and of the azide **mPEG-N_3_** used in their preparation.

The azide functional methoxypoly(ethyleneglycol) (**mPEG-N_3_**) (PEG average M_n_ = 25700) was selected by Dincer and co-workers to prepare the symmetrical and asymmetrical PEGylated zinc(II) phthalocyanines **ZnPc35** and **ZnPc36** (Figure 4) using the adequate tetra and mono terminal alkynyl substituted ZnPc [41]. The coupling between both components was performed using CuBr and PMDETA. The results showed that the introduction of the four PEG units improved the photophysicochemical properties of **ZnPc35** when compared with those of its precursor and with **ZnPc36**, namely in terms of water solubility, ability to generate ^1^O_2_ and stability. As a result of such properties they can be considered as potential PDT candidates.

**Scheme 20:**
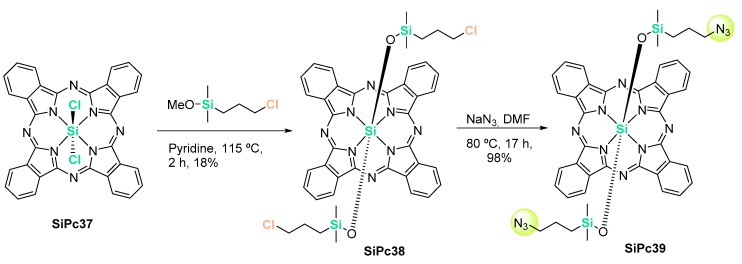
Synthesis of the azide-functionalized silicon phthalocyanine **SiPc39** used to graft polymer brushes containing acetylene pendant groups.

In 2017, Zapotoczny and co-workers reported the synthesis of a novel azide-functionalized silicon phthalocyanine **SiPc39** (Scheme 20) [42]. This Pc was then grafted with polymer brushes containing acetylene pendant groups via the CuAAC approach, using CuBr and PMDETA. The synthetic methodology to **SiPc39** was similar to the one mentioned in Scheme 13, but it involved the base-catalyzed ligand exchange of the chlorine atoms in silicon(IV) phthalocyanine dichloride **SiPc37** with 3-chloropropyldimethylmethoxysilane and then reaction with NaN_3_. The polymer brushes with acetylene side groups were obtained by surface-initiated photoiniferter-mediated polymerization. FTIR, quartz crystal microbalance, and atomic force microscopy were used to confirm the coupling of **SiPc39** with the brushes. The photophysical properties of the phthalocyanine **SiPc39** were not affected after the mentioned conjugation taking place, as evidenced by UV-Vis absorption and emission spectroscopy.

**Scheme 21:**
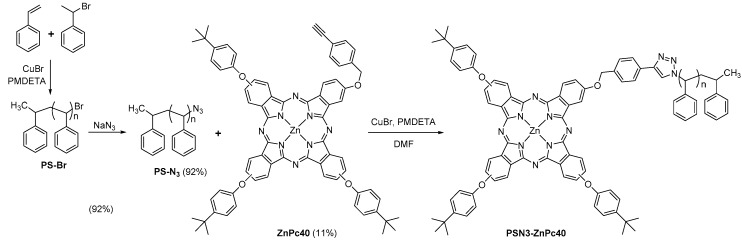
Synthetic strategy used to immobilize **ZnPc40** in the azido polystyrene **PS-N_3_**.

In 2017, Gül and co-workers selected the zinc phthalocyanine **ZnPc40** with three *tert*-butylphenoxy groups and one 4-ethynylbenzyloxy moiety to be coupled to azido polystyrene PS-N_3_ under CuAAC conditions (CuBr/PMDETA) (Scheme 21) [43]. The A_3_B-type Pc **ZnPc40** was obtained through the condensation of 4-(4-ethynylbenzyloxy)phthalonitrile and 4-(4-*tert*-butylphenoxy)phthalonitrile in 2-dimethylaminoethanol (DMAE) and the polymer was obtained from the azidation of the bromo-terminated polystyrene (PS-Br).

### 2.7. Phthalocyanines for the Development of New Electrodes

**Scheme 22:**
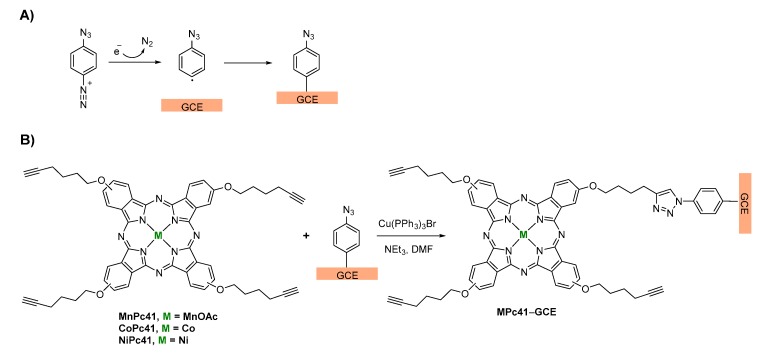
Schematic representation of the grafting of the azidobenzene diazonium salt in GCE (**A**) and of the click reactions with **MPc41** (**B**).

In the last decades, different methods and materials have been used to develop novel electrochemical sensors. According to the literature data, the modification of electrodes and their conjugation with metallophthalocyanines via the CuAAC approach did improve the stability of the sensors. In this section, it will be presented some examples of electrodes containing phthalocyanines able to recognize different analytes, such as metal ions (Hg(II), Pb(II), Cu(II)), hydrazine and hydrogen peroxide. In general, the approaches used Pcs with alkynyl units that were grafted into electrodes bearing the azido component (Scheme 22).

In 2016, Nyokong and co-workers reported the immobilization of **MnPc41** bearing terminal alkynyl units (hex-5-ynyloxy) [44] in glassy carbon electrodes (GCE) modified with the required azidobenzene component (Scheme 22B) [45]. The graft of the azido component in GCE was performed by electrodeposition of the azidobenzene diazonium salt (scanning from +0.5 V to −1 V for five cycles) (Scheme 22A) and the click reaction was performed by immersing the grafted electrode into a solution of DMF containing **MnPc41**, Cu(PPh_3_)_3_Br and triethylamine. The authors observed that the novel electrode was an efficient and robust sensor for hydrazine, showing a sensitivity of 27.38 µA mM^−1^ and a LOD of 15.4 × 10^−12^ mol dm^−3^.

Soon afterwards, the same group extended the study to electrodes prepared with the same Pc but coordinated with Co(II) and Ni(II) ions (Scheme 22) and in the case of hydrazine they found LOD values of 6.09 µM and 8.69 µM for **CoPc41-GCE** and **NiPc41-GCE** respectively, while the sensitivity was 51.32 µA mM^−1^ and 111.2 µA mM^−1^ [46].

**Figure 5:**
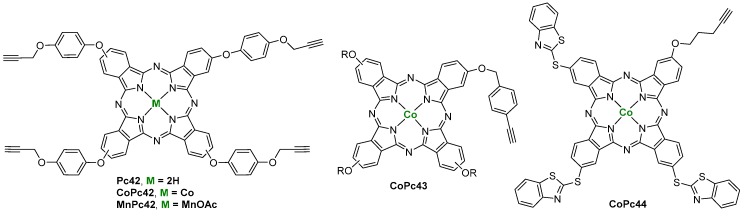
Structures of **Pc42**, **MPc42**, **CoPc43**, and **CoPc44**.

In the same year, the group selected **Pc42** with (propargyloxy)phenoxy units and its Co(II) and Mn(III) complexes to anchor in gold electrodes functionalized with the phenylazide component for the electrocatalytic detection of hydrogen peroxide (Figure 5) [47]. From the electrocatalytic studies a sensitivity value of 0.134 µA mM^−1^ cm^−2^ was obtained for the electrode clicked with **MnPc42** and 0.242 µA mM^−1^cm^−2^ for the electrode clicked with **CoPc42**.

In another contribution, the authors reported the immobilization of **CoPc43** with 4-ethynylbenzyloxy units (Figure 5) in glassy carbon electrodes modified with azide molecules and found that the platform is a good probe for hydrazine, with good catalytic rate constant (8.45 × 10^3^ M^−1^ s^−1^) and low LOD (3.28 μM) [48].

The same group also reported that the asymmetrical alkyne cobalt(II) phthalocyanine **CoPc44** after being immobilized in glassy carbon electrode (GCE) modified with the azido component, showed favorable sensitivity and lower LOD for Hg(II), Cu(II), Pb(II), and Cd(II) ions [49]. Detection limits of 81.94, 327.71, 55.87 and 347.06 nM and sensitivity of 866.23 ± 5.48, 215.82 ± 2.16, 1979.48 ± 11.47 and 204.50 ± 1.10 μA/mM were found for Hg(II), Cu(II), Pb(II), and Cd(II), respectively.

### 2.8. Click Chemistry as a Tool for New Architectures Based on Phthalocyanines

**Scheme 23:**
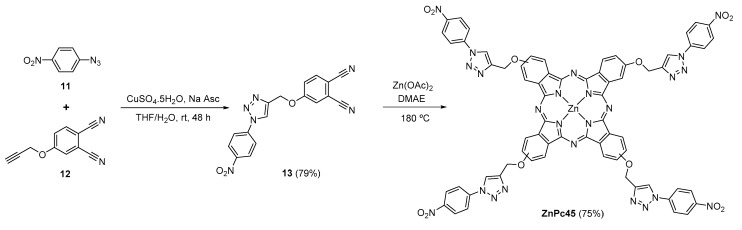
Synthetic procedures used in the preparation of **ZnPc45**.

Nyokong and co-workers have studied the non-linear optical (NLO) properties of **Pcs** combining the effects of NO_2_ and triazole linkers in their peripheral positions [50]. The synthetic access to the 4-nitrophenyl-1,2,3-triazole Pc **ZnPc45** involved the cyclotetramerization of the clickable phthalonitrile **13** which was obtained from 4-nitrophenylazide (**11**) and 4-(prop-2-ynyloxy)phthalonitrile (**12**) (Scheme 23). The click reaction was carried out in THF/H_2_O using CuSO_4_·5H_2_O as a catalyst and sodium ascorbate as the reducing agent; the cyclotetramerization of the phthalonitrile was performed in the presence of zinc acetate and DMAE. The NLO studies showed that **ZnPc45** presented better optical limiting behavior than the tetra-substituted alkynyl zinc(II) phthalocyanine selected as a potential precursor of **ZnPc45**; an improvement in triplet quantum yields and lifetimes was also observed.

**Figure 6:**
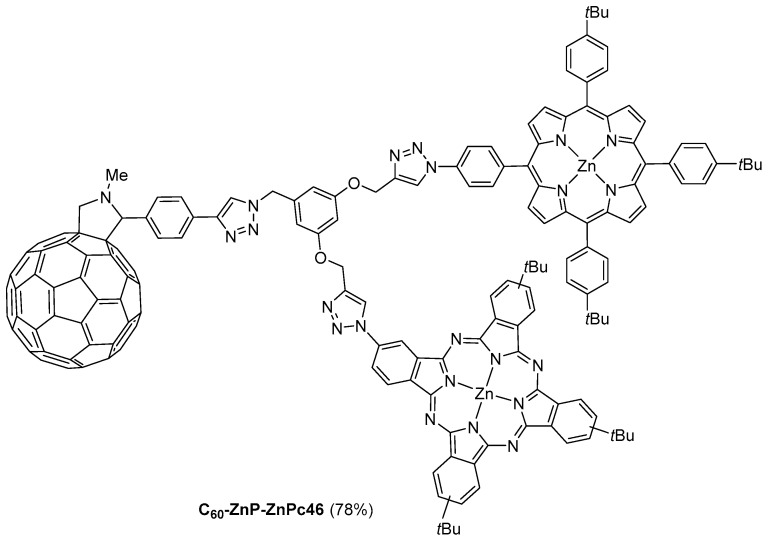
Structure of C_60_-ZnP-ZnPc46 triad.

**Scheme 24:**
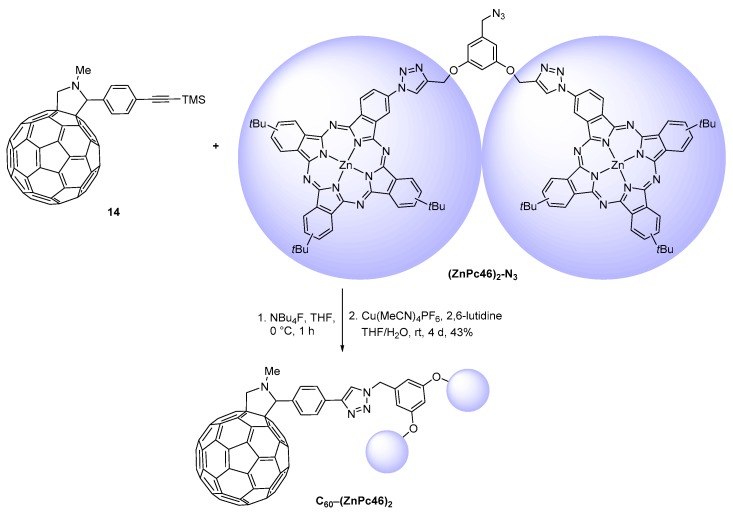
Synthetic strategy used for the preparation of the **C_60_-(ZnPc46)_2_** triad.

Campidelli and co-workers explored the CuAAC approach to obtain the fullerene-phthalocyanine **C_60_-(ZnPc46)_2_** and fullerene-porphyrin-phthalocyanine triads **C_60_-ZnP-ZnPc46** (Figure 6) and evaluated their self-assembly properties [51,52]. The azide dendron **(ZnPc46)_2_-N_3_** was also obtained using the CuAAC approach and was coupled to the alkynyl fullerene **14** after its deprotection with tetrabutylammonium fluoride with Cu(MeCN)_4_PF_6_ and 2,6-lutidine (Scheme 24). The study showed that only weak interactions were detected for this system and the electrochemical assays suggested a better interaction of the C_60_ moiety with the ZnP-ZnPc dendron. Additionally, the AFM and SEM analysis showed that these conjugates, when deposited on Si/SiO_2_ surfaces, were organized in nanofibrils of ca. 4 to 8 nm of diameter. 

**Figure 7:**
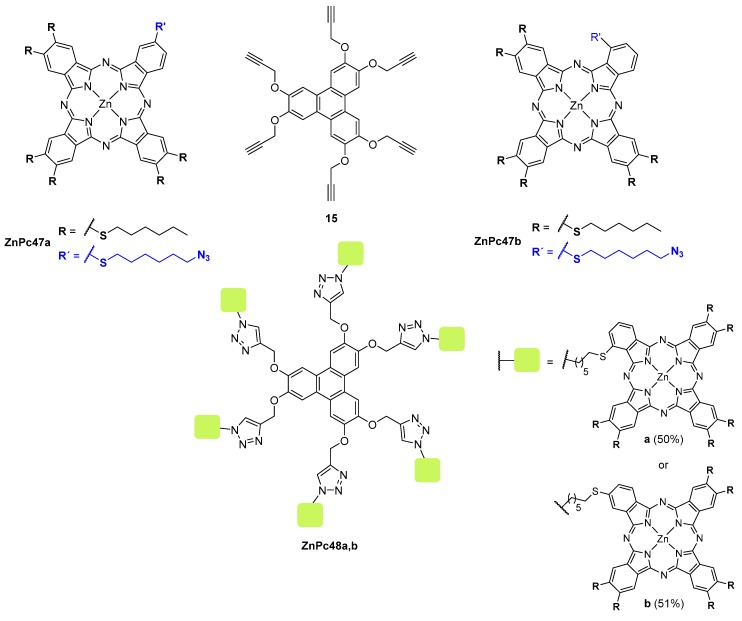
Structures of the compounds used in the preparation of the Pc derivatives **ZnPc48a**,**b**.

Gürol and co-workers prepared the asymmetric zinc(II) phthalocyanines **ZnPc47a**,**b** with six hexylthia chains on peripheral positions and one 6-azido-hexylthia chain either on peripheral (a) or non-peripheral (b) positions to be assembled to the alkyne-functionalized triphenylene core **15** in the presence of CuI (Figure 7) [53]. The obtained triazole derivatives **ZnPc48a**,**b** were tested to detect volatile organic compounds after being deposited on the surface of acoustic wave transducers via the electrospraying method. The sensorial response for acetone, ethanol, hexane, isoprene, chloroform and toluene, which can be used as biomarkers of lung cancer, was determined. Compound **ZnPc48a** showed a higher sensitivity for toluene and ethanol vapors. The obtained results confirmed the possibility to use Pc-based surface acoustic wave sensors for medical diagnosis.

**Scheme 25:**
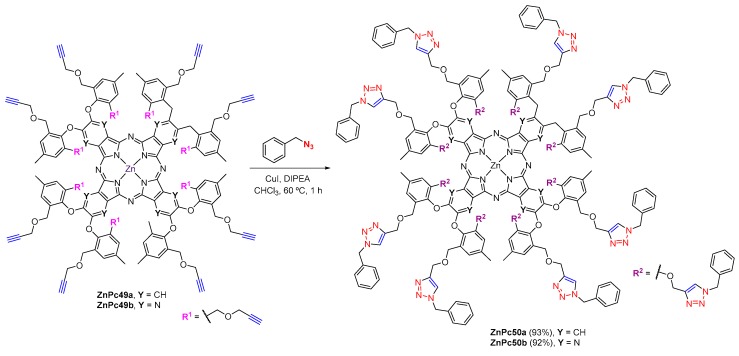
Synthetic access to **ZnPc50** from **ZnPc49**.

Recently, Makhseed and co-workers described the synthesis of the zinc(II) phthalocyanine **ZnPc49a** and of its azaphthalocyanine analogue **ZnPc49b** with multivalent propargyl moieties as new building blocks that prevent the macrocyclic planar cores from self-associating in solution or in the solid state [54]. The authors investigated the utility of these platforms in the CuAAC reaction with benzyl azide, using CuI and DIPEA in refluxing chloroform (Scheme 25). The photophysical characterization of the adducts **ZnPc50** in DMF allowed to conclude that they were exclusively in the monomeric form and the photophysical parameters were not affected by the formation of aggregates.

## 3. Subphthalocyanines

**Scheme 26:**
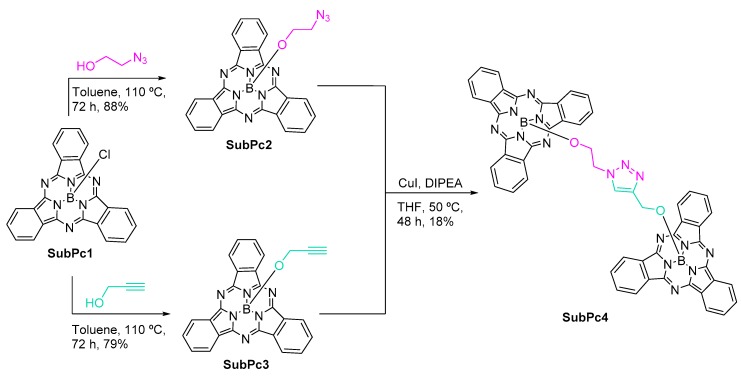
The synthetic strategy used in the preparation of subphthalocyanine **SubPc4**.

Chloroboronsubphthalocyanines have structural analogy to phthalocyanines and are also being explored in different fields, namely as chemical sensors and in electronic molecular devices. Durmus and co-workers used the monomeric subphthalocyanines **SubPc2** (bearing an azido group) and **SubPc3** (bearing a propargyl group) to afford the dimer **SubPc4** via the CuAAC approach (Scheme 26) [55]. The precursors were prepared by a ligand exchange reaction using azidoethanol or propargyl alcohol and the commercially available chlorosubphthalocyanine **SubPc1**. The synthesized subphthalocyanine dimer showed quite good solubility in most common organic solvents; the decrease in the fluorescence quantum yield and singlet oxygen generation observed after dimerization was justified as being due to the self-quenching between both subphthalocyanine units.

## 4. Porphyrazines

As already stated, porphyrazines (tetraazaporphyrins) have already been studied by many scientists interested in looking for synthetic methods leading to such compounds and for their potential applications. In general, such compounds are obtained from the cyclotetramerization of adequate dinitrile derivatives.

Eichhorn and co-workers [56] reported that azide-substituted porphyrazines with trimethylene and hexamethylene spacers and acetylene derivatives with trimethylene spacers displayed columnar mesophases (temperatures ranging from 30 °C to 110 °C) suffered an efficient cross-linking after being thermally activated. The authors found that the structure of the mesophase was not affected by the process.

**Scheme 27:**
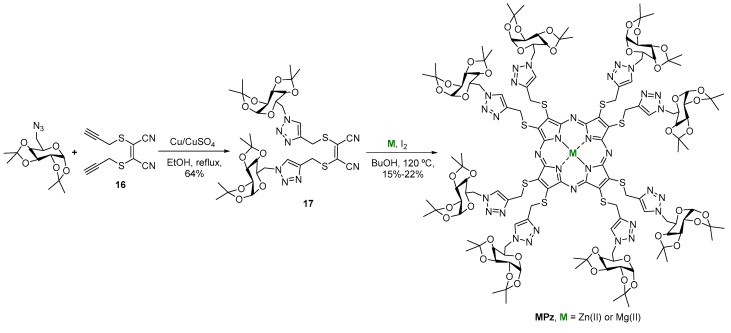
The synthesis of porphyrazines **MPz**.

In 2016, Gök and co-workers reported the synthesis of magnesium(II) and zinc(II) porphyrazine complexes (**MPz**) containing galactose moieties by the cyclotetramerization reaction of the adequate galactose dinitrile **17** (Scheme 27) [57]. Two CuAAC reactions were used to construct this dinitrile and the most straightforward one involved the conjugation of the alkynyl component **16** with 6-azido-6-deoxy-1,2:3,4-di-*O*-isopropylidene-α-D-galactose in ethanol, in the presence of Cu/CuSO_4_. The authors verified that the deprotection of the sugar units in **MgPz** with aqueous TFA led to a water-soluble porphyrazine.

## 5. Final Remarks

The significance of the CuAAC reaction to afford new systems based on phthalocyanines and related compounds like subphthalocyanines and porphyrazines is highly demonstrated in recent literature data. Most of the efforts have been related to phthalocyanines; that is probably due to the more facile strategies to obtain the adequate azide or alkynyl components and also to their already known potentiality for being used in important fields like electrocatalysis and as optical devices and chemosensors. The significance of the products obtained from the conjugation of porphyrinoids with different systems, like graphene oxide, carbon nanotubes, silica nanoparticles, gold nanoparticles, quantum dots or carbohydrates, is clearly demonstrated in this review.

## Abbreviations

ATRP—atom transfer radical polymerization

AuNPs—gold nanoparticles

CNT—carbon nanotubes

CuAAC—copper(I)-catalyzed alkyne-azide cycloaddition

DBU—1,8-diazabicyclo(5.4.0)undec-7-ene

DCC—*N*,*N*’-dicyclohexylcarbodiimide

DIAD—diisopropyl azodicarboxylate

DIPEA—*N*,*N-*diisopropylethylamine

DMAE—2-dimethylaminoethanol

DMAP—4-(dimethylamino)pyridine

DMF—*N*,*N*-dimethylformamide

DMSO—dimethyl sulfoxide

GCE—glassy carbon electrodes

HT29—human colon adenocarcinoma cells

LOD—limit of detection

mPEG—methoxypoly(ethylene glycol)

MSN—mesoporous silica nanoparticles

MWCNT—multiwalled carbon nanotube

npAu—nanoporous gold

OFET—organic field effect transistors

OPV—organic photovoltaic

Pc—phthalocyanine

PDI—photodynamic inactivation of microorganisms

PDT—photodynamic therapy

PEG—polyethylene glycol

PMDETA—*N*,*N*,*N*’,*N*’,*N*”-pentamethyldiethylenetriamine

PMMA—poly(methyl methacrylate)

PMMEM—poly(methyl methacrylate-*co*-2-(2-bromoisobutyryloxy)ethyl methacrylate) copolymer

Pz—porphyrazine

QD—quantum dot

rt—room temperature

rGO—reduced graphene oxide

SAM—self-assembled monolayer

SiNP—silica nanoparticle

SubPc—subphthalocyanine

SWCNT—single wall carbon nanotube

TBAF—tetrabutylammonium fluoride

TBTA—tris[(1-benzyl-1*H*-1,2,3-triazol-4-yl)methyl]Fuamine

TEA—triethylamine

TEG—triethylene glycol

TFA—trifluoroacetic acid

THF—tetrahydrofuran

TMS—trimethylsilyl

UV-Vis—ultraviolet-visible
